# Impact of booster vaccination on COVID-19 outcomes in Portuguese population aged 80 or more years old: Irina Kislaya

**DOI:** 10.1093/eurpub/ckac129.666

**Published:** 2022-10-25

**Authors:** I Kislaya, AP Rodrigues, S Silva, AJ Santos, C Matias Dias, B Nunes, A Machado

**Affiliations:** 1 Department of Epidemiology, National Institute of Health Doutor Ricardo Jorge, Lisbon, Portugal; 2 Public Health Research Centre, Universidade NOVA de Lisboa, Lisbon, Portugal; 3 Comprehensive Health Research Centre, Universidade NOVA de Lisboa, Lisbon, Portugal

## Abstract

**Background:**

Vaccination is essential to control SARS-CoV-2 transmission and complications. The study aimed to estimate the number of SARS-CoV-2 infections, COVID-19 hospitalizations and deaths averted by booster vaccination in Portuguese population aged 80 or more years old.

**Methods:**

We developed an ecological study for the period of the Omicron variant of concern predominance (week 2 to week 16, 2022). Data on vaccine coverage and effectiveness, and number of events of different severity reported to the national COVID-19 surveillance system were used to estimate the number of averted events, prevented fraction and number needed to vaccinate. Uncertainty intervals (UI) were obtained using Monte Carlo simulations.

**Results:**

By week 2 2022, vaccination coverage in the target population reached 91.2%. Booster vaccine effectiveness was 4.1% (CI95%: -0.1 to 9.0), 87.5% (CI95%: 84.9 to 89.7) and 83.2 (CI95%: 80.3 to 85.7) against infection, hospitalization and death, respectively. During the study period, 70862 SARS-CoV-2 infections, 2697 COVID-19 hospitalizations and 2106 deaths were reported. Booster vaccination averted 2731 (UI95%: -298 to 5838) infections, 10629 (UI95%: 9173 to 12127) hospitalizations and 6608 (UI95%: 5725 to 7546) COVID-19 related deaths among individuals aged 80 years or more resident in Portugal. Prevented fractions were 3.7% (UI95%: 0 to 7.6%), 79.7% (UI95%: 77.3 to 81.7%) and 75.8% (UI95%: 73.2 to 78.1%), respectively. It would require to vaccinate 59 individuals (UI95%: 52 to 69) to prevent one hospitalization and 94 individuals (UI95%: 82 to 109) to prevent one death in the target population.

**Conclusions:**

The booster vaccination strategy had considerable impact on preventing severe outcomes in the Portuguese population aged 80 and more years old.

**Key messages:**

• High vaccine coverage combined with high vaccine effectiveness resulted in considerable reduction of severe COVID-19 outcomes.

• Information on number of outcomes of different severity levels averted by COVID-19 booster vaccination allows to strength public health communication.

